# Detection of Illegal Race Walking: A Tool to Assist Coaching and Judging

**DOI:** 10.3390/s131216065

**Published:** 2013-11-26

**Authors:** James B. Lee, Rebecca B. Mellifont, Brendan J. Burkett, Daniel A. James

**Affiliations:** 1 Faculty of Engineering, Health, Science and Environment, Charles Darwin University, Darwin NT 0909, Australia; 2 Faculty of Science, Health, Education and Engineering, University of the Sunshine Coast, Maroochydore, QLD 4558, Australia; E-Mails: RMellifo@usc.edu.au (R.B.M.); BBurkett@usc.edu.au (B.J.B.); 3 Centre for Wireless Monitoring and Applications, Griffith University, Nathan, QLD 4122, Australia; 4 Centre of Excellence for Applied Sports Science Research, Queensland Academy of Sport, Queensland Sports and Athletics Centre Nathan Queensland 4111, Australia; E-Mail: dan@qsportstechnology.com

**Keywords:** athletics, gait, inertial sensor, Olympics, step

## Abstract

Current judging of race walking in international competitions relies on subjective human observation to detect illegal gait, which naturally has inherent problems. Incorrect judging decisions may devastate an athlete and possibly discredit the international governing body. The aim of this study was to determine whether an inertial sensor could improve accuracy, monitor every step the athlete makes in training and/or competition. Seven nationally competitive race walkers performed a series of legal, illegal and self-selected pace races. During testing, athletes wore a single inertial sensor (100 Hz) placed at S1 of the vertebra and were simultaneously filmed using a high-speed camera (125 Hz). Of the 80 steps analyzed the high-speed camera identified 57 as illegal, the inertial sensor misidentified four of these measures (all four missed illegal steps had 0.008 s of loss of ground contact) which is considerably less than the best possible human observation of 0.06 s. Inertial sensor comparison to the camera found the typical error of estimate was 0.02 s (95% confidence limits 0.01–0.02), with a bias of 0.02 (±0.01). An inertial sensor can thus objectively improve the accuracy in detecting illegal steps (loss of ground contact) and, along with the ability to monitor every step of the athlete, could be a valuable tool to assist judges during race walk events.

## 1. Introduction

**Figure 1.**
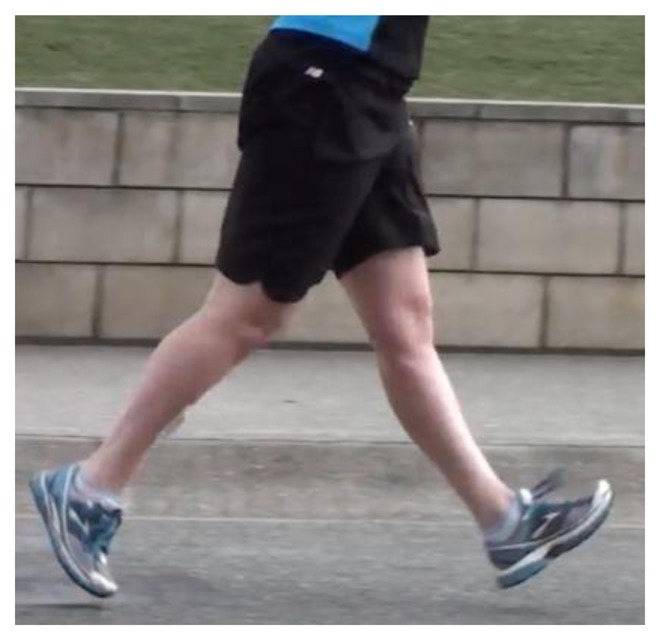
An athlete shown with no ground contact, therefore an illegal walking action.

Race walkers have a gait style that is unique when compared to normal walking. This different style results in variations in physiological and gait characteristics, when compared to other aerobically trained athletes. This includes an increase in walking speed before transition to running [1]. Race walking has a long (and at times contentious) history in international competition. The sport was first included as a permanent Olympic event in 1908 and has been included in the International Association of Athletics Federation (IAAF) Athletics World Championships since their inception in 1983. The event is determined by the fastest race walker. An athlete can be disqualified by breaching the rules laid out by the IAAF [2]. The most common rule breach is loss of ground contact, in other words a flight phase (Figure 1). The “Definition of Race Walking” relates to continual ground contact as explained in the first sentence of IAAF Competition Rule 230 states: “*Race Walking is a progression of steps so taken that the walker makes contact with the ground, so that no visible (to the human eye) loss of contact occurs*” [2]. Currently these decisions are based on the subjective assessment of the judge's eye, making fair assessment in this long-standing Olympic event difficult [3]. Subsequently the ongoing credibility of this international sport has been questioned by the few scientific studies carried out [3]. Researchers have been cited in popular athletics journals/publications stating this subjectivity is considered a weakness of the sport as well as from within the sport's own ranks [4].

From the events at the 2012 London Olympic Games and 2011 IAAF World Championships in Athletics, 12% of race walkers were disqualified for either loss of ground contact or a bent knee at initial contact [5]. Approximately three-quarters of the recorded reasons for disqualification were for loss of ground contact. An athlete can receive two separate warnings from different judges for either or both of the indiscretions. A third infringement results in disqualification. The current visible observation by the race judges contains two flaws. Firstly, the fastest rate a human eye can retain an image is 16 Hz, or 0.06 s. Anything faster cannot be accurately processed [6]. Therefore, if the period of flight is less than 0.06 s, this will not be accurately detected by observation alone. Additionally, at many instances during competitions, athletes race in “pack” situations. Therefore making observation difficult to assess all athletes independently *i.e.*, to clearly separate one athlete's gait from another. A theory known as “change blindness” may also cause a judge to miss illegal steps, or perceive that they are seeing an action that is not occurring [7,8]. This is particularly common if their attention is distracted from one point of focus, such as judging walkers in a pack and simultaneously checking for loss of ground contact and assessing knee angle at landing. The second flaw is the judges are only stationed at various points on a race circuit and therefore have restricted periods of assessment, especially if the course is away from a track environment. Race walk events are up to 50 km in length and so it is often impossible for the whole course to be monitored. Furthermore, only one judge can only observe one athlete at a time. The consequences of a missed or incorrect disqualification whilst competing on the world's greatest sporting stage could be devastating to the individual athlete. Additionally, typical media scrutiny judging errors can cause embarrassment to the governing body.

Both problems may be overcome by the use of microtechnology. Inertial sensors are unobtrusive, lightweight, wireless, inexpensive and commercially available which makes them an attractive option for field-based research [9]. Inertial units containing triaxial accelerometers have been shown to have the capability to measure spatio-temporal kinematics of gait in walking and running [10–13]. Furthermore, research has demonstrated an inertial sensor's capability to discriminate between walking and running gaits [14]. Once validated for the unique race walking gait profile, a single inertial sensor worn by each competitor would not only remove the subjectivity in judging loss of ground contact, but also enable each and every step taken throughout the event to be monitored. Additionally, this novel technique could be utilized in the athlete's training sessions to provide race-standard feedback on any illegal technique. To address these issues the aim of this study was to determine whether a single inertial sensor could detect the loss of ground contact (flight) in race walkers.

## 2. Methods

Seven race walkers (five males, two females) volunteered for the study (Table 1). Participants signed a written informed consent, in accordance with the Statement on Human Experimentation by the National Health and Medical Research Council of Australia. The Human Research Ethics Committee of the University of the Sunshine Coast approved the study.

Data was captured at a tartan Olympic standard athletics track by two methods: (1) a high-speed camera (Troubleshooter, model TSHRMM, Fastec Imaging, San Diego, CA, USA). Calibration of the high-speed camera used a wand (length = 1 m) that was placed in the center of the trial lane and seen in the center of the capture window and recorded; (2) the derivative of a previously developed inertial system [15] was used as the inertial sensor in these tests. The gravitational method of calibration was used as described by Lai *et al.* [16]. Previously this sensor has been validated for ground contact events in human locomotion [17] against insole based pressure sensors. The sensor system uses a RISC-based microprocessor using an event driven real time operating system, sensor data was sampled at 100 Hz for three orthogonal channels of acceleration and filtered with a low pass filter at a 20 Hz cutoff frequency, data is stored locally and downloaded serially using a USB interface.

**Figure 2.**
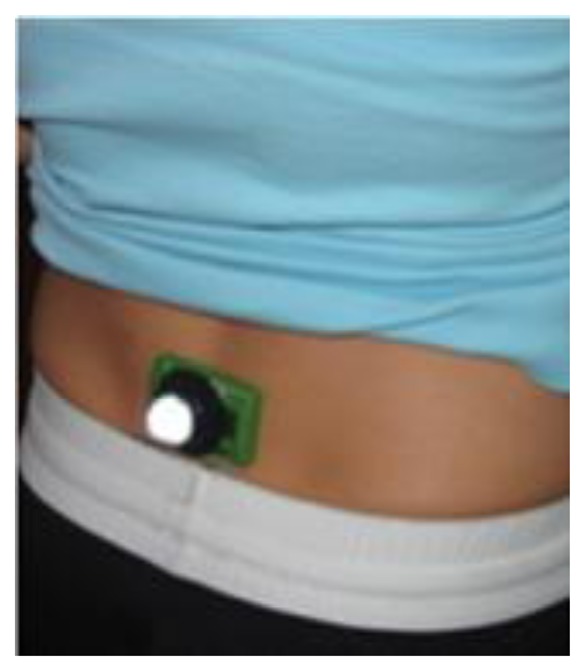
Typical sensor placement at S1 of the sacrum.

The inertial sensor was placed directly on the skin over the S1 vertebra of the sacrum of each of the volunteers (Figure 2). The camera was positioned perpendicular to the athletics track 4.65 m from the center of the trial lane with a 2.27 m capture window. The camera captured data at 125 Hz and the inertial sensor captured data at 100 Hz. Following each athlete's individual warm up protocol, instructions were provided by their coach to perform four trials of each of three race walking styles, namely: (a) walking legally at submaximal racing pace; (b) walking illegally at submaximal race pace, that is with a flight phase, and (c) walking legally at race pace. Due to outcomes that were presented in the data *i.e.*, athletes walking illegally even when instructed to walk legally (conditions a & c), it was decided to group all three conditions into one for statistical analysis. This was carried out because the study's aim was to determine the effectiveness of inertial sensors to detect flight. In other words, when steps were legal or illegal as seen in the high-speed camera data, were these legal or illegal in the inertial sensor data? Reasoning for describing the three conditions were kept to highlight that performing illegal steps might be difficult to overcome. There was at least 50 m lead-in distance and three minutes recovery between each walk to minimize any influence from the previous walk. This regime also allowed the athletes' own perceptions of how they walk to be assessed.

Analysis of high-speed camera data was carried out by observation of captured sessions. This involved identifying video frames where initial ground contact was made and where the contralateral toe off occurred. The high-speed camera-capture method identified 57 illegal steps with flight and 23 legal steps for a total of 80 steps. The data on these steps were compared with the sensor-captured data on the same steps. The data were converted to time (in seconds) of loss of ground contact by the following the equation:

t=(Σn)/Hz

where *t* = time in seconds; Σ*n* = the sum of frames between gait events of toe-off of one foot and heel strike of the contra-lateral foot; *Hz* = the frequency of the capture system. Mean ± SEM time of continuous ground contact was calculated for both sets of data.

For the inertial sensor data, the Vertical Acceleration Step Cycle method was used [14]. Based on research by Little *et al.* [14], it was hypothesized that flight would occur when heel strike seen in anteroposterior acceleration data coincided with, or just after, the bottom most point in vertical acceleration data. Due to toe off kinematics coinciding at or around the time of heel strike [17], determination of heel strike timing alone was carried out, as reported by Little *et al.* [14]. The ground contact force transmission offset of heel strike relative to vertical acceleration was found by assessing the data using limits of agreement (Typical Error of the Estimate) analysis [18]. Assessing this agreement found the offset to be 0.03 s. While flight time was measured in the high-speed camera data and used as the comparison in the agreement analysis, only the timing of heel strike was measured in the inertial sensor data. Due to the research aiming to determine the effectiveness of an inertial sensor in detecting legal or illegal steps, flight time was not necessary. Once heel strike occurred after the 0.03 s threshold, flight was deemed to have occurred.

For high-speed camera data, if the toe-off and heel-strike events coincided in the same capture frame, timing equaled the threshold. If there was a period of double support, time was reported as positive data. Where an illegal walk was indicated (loss of ground contact), time was reported as negative data. Testing for operator error in identifying heel strike and toe off gait events in the high-speed camera data used the measures of reliability method [19] with a 95% confidence limit (CL). The process involved randomly selecting 50 race walking strides and identifying the respective video frame number in which each of the gait events occurred. To test for consistency, this was repeated on the same data sets three times. To minimize any memorizing effect, the retests were carried out over consecutive days.

Statistical analysis using a limits of agreement method was carried out using Pearson's correlation, Typical Error of the Estimate (magnitude of inertial sensor error relative to the high-speed camera), and mean bias (positive result would indicate the mean occurred before the threshold, a negative result would indicate the mean occurred after the threshold) [18]. These measures were used to validate the inertial sensor measures against the high-speed camera benchmark.

## 3. Results and Discussion

The total gait events tested were 300 *i.e.*, 50 heel strikes and 50 toe offs, repeated three times. Six heel strikes and eight toe offs made up the 14 wrong event identifications. The reliability test found a typical error of 0.24 (lower CL: 0.20, upper CL: 0.31) and an intraclass correlation of 0.99. This confirmed operator error as minimal. The actual error was no more than a single frame in each of the 14 event identifications that were found to be incorrect.

The statistical analysis of the race walking data found the Typical Error of the Estimate was 0.02 (95% confidence limits; 0.01 to 0.02) s, and a mean bias of 0.02 (±0.01). There were strong correlations (*r* = 0.67) of detecting legal and illegal flight measured by both methods. The correlation result indicates that the data did not always match the exact flight time, or ground contact time. However, actual flight was not the primary objective. The main focus was to determine the effectiveness of discriminating legal and illegal steps. This was seen to be highly effective with 34% more accuracy shown than scientific tests of race walking technical-official judgments [3].

**Figure 3.**
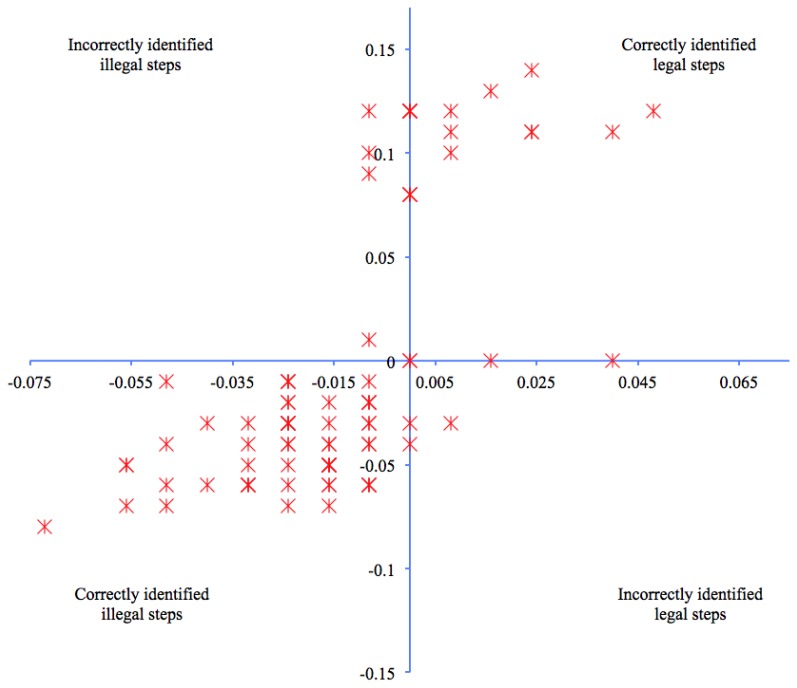
Inertial sensor and high-speed camera comparison of legal and illegal steps. X axis represents step timings captured by the high-speed camera. Y axis depicts inertial sensor step timings. Positive data from either system indicate legal steps. Negative data represents illegal steps. Therefore the top right and bottom left quadrants show inertial sensor data in agreement with high-speed camera data for respective legal and illegal steps. The top left quadrant indicates illegal steps identified in the camera data and not detected by the sensors. The bottom right quadrant depicts steps in the camera data that were shown to be legal and deemed illegal in the inertial sensor data. Markers that are slightly darker than other data indicate multiple gait events occurring at that point in the plot.

Of the 80 steps analyzed, observation of the high-speed camera datasets showed 57 as illegal, of which four were misidentified by the inertial sensor (Figure 3). All four missed illegal steps had 0.008 s of loss of ground contact. Twenty-three legal steps were identified by the high-speed camera, of these; three were identified as illegal steps by the inertial sensor. Two of the three legal steps wrongfully identified as being illegal showed heel strike and toe off occurring in the same capture frame by the high-speed camera, that is 0.00 s loss of ground contact. The third incorrect identification was incorrect by 0.03 s. This level of detection demonstrated the inertial sensors' ability to correctly identify the illegal or legal race walking steps 91% of the time.

It is worth noting that when the athletes were instructed to perform as they do during a race (condition c), no one walked with constant ground contact. Furthermore, several of the athletes did not walk legally when asked to do so at submaximal pace. Both systems detected these occurrences.

When compared to the high-speed camera, the inertial sensor was 91% accurate at correctly identifying a legal or illegal step, as defined by a loss of contact with the ground. However, the flight or stance time for the seven incorrectly identified steps was 0.008 s, which is considerably less than what the human eye has been found to detect. The fastest action that can be visually processed accurately is 0.06 s [6]. One previous study assessed biomechanical characteristics of race walking gait [20]. The authors reported that athletes tested lost ground contact up to 0.04 s, which would not be detectable by judges. Another study compared race walk judge's visual accuracy and found that steps with flight times less than 0.05 s were not deemed as disqualifiable by the judges [3]. The study's authors grouped illegal steps that were identified using high-speed camera data into two categories: flight times less than 0.05 s and flight times greater than 0.05 s. Errors made by judges would almost be certain when the period of flight is considerably less than what can be detected by human sight. This was demonstrated from the analysis of these two sub-groupings. In the first category, no illegal steps were identified. Judges still only managed an accuracy rate of 57% in the second of the two categories (greater than 0.05 s flight time). Additionally, each identified illegal step was not distinguished by another judge, indicating subjectivity in the judging. By comparison, the current study found inertial sensors to have an accuracy of 91% at much less flight times as seen in the high-speed camera data. The previous study only reported missed illegal steps. When only considering illegal steps, the inertial sensor accuracy rate increased to over 96%, or 3 legal steps identified as illegal *i.e.*, steps in the bottom right quadrant (including two positioned on the Y axis) (Figure 3). The current authors wish to express concern that any wrongful identification of a legal step as being illegal to be unfair on an athlete walking correctly. Further research is required to determine whether refinement of the detection process could improve false identification of legal steps. No previous research could be found in the literature that investigated the wrongful identification of legal steps by judges. Outcomes of such a study may give better indications to whether inertial sensors are more or less accurate relative to the wrongful identifications of legal steps. Nonetheless, raising the accuracy from a level of less than 60% accurate to one that is in excess of 90% must ultimately benefit athletes.

The current study indicates that a single inertial sensor can be more accurate at detecting illegal steps than by the naked eye. However, the authors are not implying that inertial sensors replace race walking judges, but the units be used as a tool by race officials as a judging aid. The benefit as a judging tool is that the units offer the possibility of monitoring, real-time, each step that is taken by an athlete wearing a unit. Other studies have reported the effectiveness of inertial sensors for delivering real-time data during human movement analysis [21,22]. A centrally based computer could monitor all competitors by receiving data from inertial sensors being worn. Any possible illegal steps performed by an athlete could be detected. That person can be bought to the attention of the judges for closer scrutiny. Evidence shown by researchers such as Knicker and Loch [3] indicate the judging the legality of race walking using only the naked eye can result in two possible outcomes: firstly: the judge has to be conservative in the process to be sure of an illegal action, and therefore may miss flight within illegal strides; secondly: attempt to second-guess what is happening, which also may result in inaccurate judging. The combination between the established method of race walk judging and the use of inertial sensors may have merit.

Further to the limitations of visual judging, one could even consider what might happen at the limit of eye processing. A judge might make presumptions in what is occurring. When attempting to observe race walkers in pack situations, a judge might not be able to process actions effectively due to change blindness. Comments by previous researchers explain how a person can observe an action and perceive a movement that might or might not be occurring [7,8]. This type of perception deficiencies due to eye processing limits would likely leave a judge to make subjective decisions. Using microtechnology to assist in judging of race walkers may benefit the image of the sport in general and fairness for its athletes in particular.

The ability of inertial sensors to measure every step taken by the athlete would be beneficial. This gives a justifiable argument to promote the use of a single, small inertial sensor to support the current method of judging in this Olympic event. Due to the unobtrusive nature of the sensor, the technology may also be used as a training tool for athletes [23]. By wearing the device during training any illegal technique could be detected and subsequently addressed. This study has shown that some athletes did not realize they lost ground contact, *i.e.*, walking illegally even though instructed to walk legally.

Future research may include: (1) investigating the effectiveness of monitoring the effects of fatigue; (2) refinement of the inertial system to greater than the 91% accuracy reported here. Sensors have been shown to detect changes in symmetry [24] a known consequence of fatigue [25]. As the device measures acceleration with respect to time, this novel application may also be able to identify any relationship between fatigue and illegal walking [26]. Further refinement of this system may improve accuracy of the inertial sensor compared to a high-speed camera in correctly identifying race walking steps. This is most important in regards to the wrongful identification of legal steps as illegal. While it may be considered that the system reported here might not be appropriate to measure flight time, that was not the intention. The intention was to investigate the effectiveness of sensors to determine whether a walker is performing legal or illegal steps. In this case, the system dramatically increases the accuracy (91%) when compared to what has been reported (57%) regarding the current system of judgment [3]. This study sets the basis for research to refine the system further in order to take the measures from the accuracy reported here towards the ideal of 100%.

## 4. Conclusions

Inertial sensors were shown to have an accuracy rate of 91% when compared to a high-speed camera. The seven incorrectly identified steps occurred with a time change considerably less than what could be detected by the human eye. This research provides an opportunity to develop an inertial sensor based tool for coaching purposes that would assist athletes who are not aware they are performing illegal steps to correct their technique. Furthermore the ability to measure each step taken by an athlete suggests it may be possible that inertial sensors could be used as a tool to assist judges monitor Olympic and international race walking events.

## Figures and Tables

**Table 1.** Characteristics of participating race-walkers.

	**Age (years)**	**Height (cm)**	**Weight (kg)**	**Experience (years)**
Mean	25	177	67	12
SD	7.3	8.2	10.2	10.5
